# Ultraviolet-C-based sanitization is a cost-effective option for hospitals to manage health care-associated infection risks from high touch mobile phones

**DOI:** 10.3389/frhs.2024.1448913

**Published:** 2025-01-13

**Authors:** David C. Cook, Matthew Olsen, Oystein Tronstad, John F. Fraser, Adrian Goldsworthy, Rashed Alghafri, Simon J. McKirdy, Lotti Tajouri

**Affiliations:** ^1^ Harry Butler Research Institute, Murdoch University, Murdoch, WA, Australia; ^2^ School of Agriculture and Environment, The University of Western Australia, Crawley, WA, Australia; ^3^ Faculty of Health Sciences & Medicine, Bond University, Robina, QLD, Australia; ^4^ Critical Care Research Group, The Prince Charles Hospital, Chermside, QLD, Australia; ^5^ Faculty of Medicine, University of Queensland, Herston, QLD, Australia; ^6^ Physiotherapy Department, The Prince Charles Hospital, Chermside, QLD, Australia; ^7^ School of Clinical Sciences, Queensland University of Technology, Brisbane, QLD, Australia; ^8^ Northside Medical School, University of Queensland, Chermside, QLD, Australia; ^9^ School of Medicine, University of Queensland, Herston, QLD, Australia; ^10^ International Centre for Forensic Sciences, Dubai Police, Dubai, United Arab Emirates; ^11^ Dubai Police Scientific Council, Dubai Police, Dubai, United Arab Emirates

**Keywords:** mobile phone sanitizer, ultraviolet-C, hand hygiene, public health, fomite, antimicrobial, cost effectiveness analysis, hospital-acquired infections

## Abstract

Mobile phones have become essential tools for health care workers around the world, but as high touch surfaces, they can harbor microorganisms that pose infection risks to patients and staff. As their use in hospitals increases, hospital managers must introduce measures to sanitize mobile phones and reduce risks of health care-associated infections. But such measures can involve substantial costs. Our objective in this paper was to consider two mobile phone risk mitigation strategies that managers of a hypothetical hospital could implement and determine which involves the lowest cost. The first strategy required all staff to sanitize their hands after every contact with a mobile phone. The second involved the hospital investing in ultraviolet-C-based mobile phone sanitization devices that allowed staff to decontaminate their mobile phones after every use. We assessed each intervention on material and opportunity costs assuming both achieved an equivalent reduction in microbe transmission within the hospital. We found that ultraviolet-C devices were the most cost-effective intervention, with median costs of approximately AUD360 per bed per year compared to AUD965 using hand hygiene protocols. Our results imply that a 200-bed hospital could potentially save AUD1–1.4 million over 10 years by investing in germicidal ultraviolet-C phone sanitizers rather than relying solely on hand hygiene protocols.

## 1 Introduction

Managing health care-associated infection (HAI) risks associated with mobile phones is particularly challenging to hospital administrators. HAIs are infections acquired during hospital care that were not present or incubating at admission (1), resulting in significant patient morbidity and mortality, prolonging the duration of hospital stays and necessitating costly diagnostic and therapeutic interventions (2). One in 10 hospitalized patients in developing countries experience HAI, and seven out of every 100 hospitalized patients in developed counties (3). In the U.S., around 1.7 million HAIs occur annually (4), costing US$10–147 billion per year (5, 6). In Australia, HAIs result in approximately two million bed days per year (7). The added cost per HAI in Europe has been estimated at €4,900, which includes additional time in hospital, antibiotics, pharmaceutical products and other necessary medical services (8).

Occurrences of HAIs continue to escalate (9), likely influenced by the increasing use of unsanitized mobile phones in health care settings (10–12). Mobile phones are high-touch microbial laden platforms that negate hand hygiene. As fomites, they harbor all classes of microbes, including viruses, bacteria, fungi and protozoa in addition to a large spectrum of antibiotic resistant and virulence factor genes (13). This makes their inclusion in the management of staff-to-patient HAI risk critical as fomite-based transmission is often neglected and is difficult to manage. Not only are the potential costs of HAIs high for the patients experiencing preventable complications; managers of health care facilities must also consider reputational risks to their businesses in the age of social media as HAIs are now used as indicators of the quality of patient care (14).

Numerous studies from all over the world have reported the presence of microbes known to cause HAI on mobile phones used by healthcare workers (HCW). For example, Brady et al. (15) found that of 105 UK HCW mobile phones tested, 96.2% showed evidence of bacterial contamination and 14.3% were contaminated with bacteria known to cause HAIs. Brady et al. (16) revealed that bacteria known to cause HAI were routinely transported into the operating environment on the mobile phones of medical staff at a medical facility in Scotland. Lee et al. (17) reported that bacteria with pathogenic potential were present on 28.6% of HCW mobile phones tested from three hospitals in South Korea. Apivanich et al. (18) tested 173 HCW mobile phones and reported a bacterial contamination rate of 100%. Qadi et al. (19) discovered microbial contamination on 87.5% of HCW's mobile phones tested in Palestine and concluded that mobile phones presented a significant epidemiologic hazard to the public. Tajouri et al. (20) identified 58 human pathogenic and commensal bacteria from 30 mobile phones of HCW in Australia. Olsen et al. (21) demonstrated in their systematic review that 45% of all phones investigated during Covid-19 did harbor SARS-CoV-2 coronavirus. Reviews of the literature on microbial contamination of mobile founds can be found in Ulger et al. (22) and De Groote et al. (23).

One strategy that hospital managers can use to mitigate the risks of staff-to-patient HAIs attributable to mobile phones is to mandate that HCW sanitize their hands after touching their mobile phones and before they interact with patients. This strategy relies on HCW complying and following appropriate hand sanitization techniques, such as those recommended by the United States Centers for Disease Control and Prevention, using either soap and water or liquid hand sanitizers (24). The benefits of hand sanitization has long been understood by medical practitioners (25), although its effectiveness depends greatly on the level of compliance (26). The costs of this strategy can be evaluated in terms of water use, soap and liquid sanitizer purchase costs, and the opportunity costs of HCW time spent cleaning their hands.

A novel alternative to hand hygiene protocols is to require ultraviolet-C (UV-C) sanitization of phones prior to entry into a health care facility and after each use. UV-C emitting technologies use light sources of approximately 265 nm germicidal waves that are directed onto surfaces for sanitization and are used in medical laboratories worldwide (13). Microorganisms have peak light absorbances at 260–265 nm (27), so absorb the UV light emitted by these devices which results in the disruption of DNA or RNA (28). Several mobile phone UV-C sanitization devices are commercially available and are sufficiently small to be installed in hospitals next to patient beds. Recent studies have demonstrated that treating mobile phones with these devices after each use greatly reduces the risk of microbial transfers to HCW hands and subsequently to patients (29–38). While relatively costly to purchase, these devices are hand-free, require minimal maintenance and generally have a long operational life. Moreover, they could potentially lower hand sanitization costs to hospitals as HCW do not (necessarily) need to wash their hands after using UV-C-sanitized mobile phones if the phone is subjected to UV-C after each use.

In this paper, we examine the cost effectiveness of hand hygiene protocols and UV-C phone sanitizers as strategies for hospitals to minimize HAI risks related to mobile phones. This involves comparing the costs of each alterative over an investment horizon and determining which is the cheapest investment option for the hospital. Because there is no data directly comparing the benefits of these two strategies in reducing HAIs, we focus our attention on their costs in this hypothetical study and assume the interventions have equal effectiveness in reducing microbial dissemination. If hospital management are motivated to minimize costs, the option identified as having the lowest cost is preferable.

Although not as widely used as cost benefit analysis, which compares the net gains produced by different interventions, cost effectiveness analysis avoids an explicit quantification of benefits (39). This is pragmatic in our case study, for the health benefits of hand washing and UV-C phone sanitization are nuanced and underexplored. To simulate the complete theoretical benefits of preventing the many microbes affected by the proposed interventions, each with their own health risks, spread characteristics, pathogenicity, and information constraints, would be a large undertaking that is unlikely to add additional information relevant to clinicians or healthcare decision makers. By assuming the health benefits of each intervention are the same, which we feel is a realistic assumption, our study is more simplistic than a benefit cost analysis but remains informative to decision-makers.

The following sections outline the model and parameters we used to determine the cost of each intervention to the hospital and the outcomes of the cost effectiveness evaluation. This incorporates model outputs, including the total cost of interventions, the net present value achieved by the hospital investing in UV-C phone sanitization devices, and the sensitivity of these results to uncertainties in model parameters. All results are given in Australian dollars unless otherwise stated.

## 2 Materials and methods

We considered a hypothetical hospital situated in an unspecified developed economy. Hospital management was faced with a decision to either continue using contemporary methods of mitigating staff-to-patient HAI risk via mobile phones using handwashing protocols or to invest in a new technology that achieves the same risk reduction using UV-C sanitization. The cost effectiveness of these investment alternatives was assessed over a planning horizon of 10 years according to material and opportunity costs. The model equations are stated below in their non-simplified form to make them easier to follow as standard units for some of the parameters required conversion (e.g., seconds to hours, hours to years, kilowatts to watts).

The material costs involved in traditional HAI risk-reduction techniques relying on hand sanitization with either soap and water or hand sanitizer are numerous. Firstly, they include water costs. The hourly cost of water used by a HCW for hand washes after mobile phone use $\left(C_{w}\right)$ was calculated as:

$$C_{w}=T.\frac{D_{sw}}{3,600}.\frac{F}{3,600}.P_{w}.(1-\;\alpha_{1})\;$$

where: *T* is the number of HCW hand touches per hour on mobile phones while on duty; $D_{\mathrm{sw}}$ is the average duration of hand washes with soap and water in seconds; *F* is the average tap flow rate in L/s; $P_{w}$ is the water price in $/L; and $\alpha_{1}$ is the proportion of HCW who routinely disinfect their phone using other methods (e.g., alcohol wipes) while on duty.

Additional material costs incurred using traditional hand washing are the costs of soap, $C_{s}$, the costs of hand sanitizer, $C_{h}$, and the costs of paper towels for hand drying, $C_{t}$. The hourly cost of soap, sanitizer and paper towels used by a HCW were calculated as:

$$C_{s}=V_{s}.\;P_{s}.T.(1-\;\alpha_{1})\;$$

$$C_{h}=V_{h}.\;P_{h}.T.(1-\;\alpha_{1})\;$$

$$C_{t}=V_{t}.\;P_{t}.T.(1-\;\alpha_{1})\;$$

where: $V_{s}$ is the volume of (liquid) soap used per wash in ml; $P_{s}$ is the price of soap in $/ml; $V_{h}$ is the volume of hand sanitizer used per wash in ml; $P_{h}$ is the price of hand sanitizer in $/ml; $V_{t}$ is the volume of paper towel used per wash expressed as the number of sheets used; and $P_{t}$ is the price of paper towel expressed in $/sheet.

The opportunity cost of time HCW spend washing their hands captures the benefits of activities forgone because of using the traditional hand hygiene option. Hourly opportunity costs per HCW associated with this intervention, $C_{o}$, were approximated as:

$$C_{o}=T.W.\left[\left(\alpha_{2}.\frac{D_{sw}}{3,600}\right)+(1-\alpha_{2}).\frac{D_{h}}{3,600}\right].(1-\;\alpha_{1})$$

where: *W* is the wage rate in $/h; $\alpha_{2}$ is the ratio of hand washes with hand sanitizer to washes with soap and water; $D_{h}$ is the average duration of a hand wash with hand sanitizer in seconds; and 1 h consists of 3,600 s.

Using our measures of $C_{w}$, $C_{s}$, $C_{h}$, $C_{t}$ and $C_{o}$, we determined the costs to the hospital over 1 year of opting for traditional hand hygiene protocols to control HAI risks from mobile phones, $TC^{hw}$, to be:

$$TC^{hw}=[B.\alpha_{3}.\;(C_{w}+C_{s}+C_{h}+C_{t}+C_{o})].8,760$$

where: *B* is the number of patient beds in the hospital; $\alpha_{3}$ is the HCW-to-patient ratio; and 1 year includes 8,760 h.

Given hospital managers would be expected to assess the cost effectiveness of the hand washing option by summing $TC^{hw}$ over a planning horizon of several years, we applied a discount rate to convert future costs to present value terms. Discounting has an increasingly erosive effect on monetary values over time, meaning that the value of one unit of cost caused today is worth more than the same unit of cost incurred a year or more in the future. Over *n* years, the present value of costs associated with the hand hygiene option to control HAIs, $PVC^{hw}$, is calculated as the sum of discounted $TC^{hw}$ in each year:

$${PVC}_{n}^{hw}=\sum_{i=1}^{n}\frac{{TC}_{i}^{hw}}{{\left(1+r\right)}^{i}}$$

where: *r* is the discount rate; and *i* is the period (year) in which costs are incurred.

The costs associated with the second investment option, using UV-C sanitization devices to treat mobile phones, also include material costs and opportunity costs. The first material cost we considered was the electricity cost, $C_{e}$. We calculated the hourly cost of electricity needed to run each UV-C phone sanitization device as:

$$C_{e}=T.E.\frac{P_{e}}{1,000}.\frac{D_{uv}}{3,600}.(1-\;\alpha_{1})\;$$

where: *E* is the power rating of a standard UV-C sanitization device in watts; $P_{e}$ is the price of electricity in $/kWh; and $D_{uv}$ is the average duration of phone sanitization using a UV-C phone sanitizer unit in seconds.

The next material cost incurred using UV-C devices we considered was the combined purchase, installation and maintenance cost, $C_{p}$. This cost, expressed on an hourly basis, was calculated as:

$$C_{p}=\left(P_{uv}.\frac{O^{-1}}{8,760}\right).(1+\alpha_{4})$$

where: $P_{uv}$ is the purchase and installation price of a single UV-C unit in $; *O* is the operational life of the device in years; and $\alpha_{4}$ is the maintenance cost expressed as a proportion of the purchase and installation costs.

We assumed that HCW would not attend to other duties while waiting for their mobile phones to be sanitized in a UV-C device, implying an opportunity cost of activities forgone. Hourly opportunity costs per HCW of using UV-C devices, $C_{\hat{o}}$, were approximated as:

$$C_{\hat{o}}=T.W.\frac{D_{uv}}{3,600}.(1-\;\alpha_{1})$$

The total costs to the hospital of opting for UV-C devices over one year, $TC^{uv}$, were therefore calculated as:

$$TC^{uv}=[B.\alpha_{3}.\alpha_{5}.\;(C_{e}+C_{p}+C_{\hat{o}})].8,760$$

where: $\alpha_{5}$ is the ratio of UV-C sanitization devices to hospital beds. We assumed as our base case that one UV-C device would be supplied for every two hospital beds but the sensitivity of results to this assumption is explored below.

Over a planning horizon of *n* years, the present value of costs associated with the UV-C device option to control HAI risks, $PVC^{uv}$, was calculated as the sum of discounted $TC^{uv}$ in each year:

$${PVC}_{n}^{uv}=\sum_{i=1}^{n}\frac{{TC}_{i}^{uv}}{{\left(1+r\right)}^{i}}.$$

Using estimates of $TC^{hw}$ and $TC^{uv}$, we calculated the net present value to the hospital of investing in UV-C devices, $NPV^{uv}$. Over a planning horizon of *n* years, $NPV^{uv}$ was calculated as:

$${NPV}_{n}^{uv}=\sum_{i=1}^{n}\frac{{TC}_{i}^{hw}-{TC}_{i}^{uv}}{{\left(1+r\right)}^{i}}.$$

Model parameters and their assumed values, drawn from the relevant literature, appear in Table 1. Using the Monte Carlo method, parameters were specified as uniform distributions with minimum and maximum values when their values were not certain, but for the number of paper towels used per hand wash a discrete distribution was used with three possible outcomes, all with the same probability of occurrence. We performed 10,000 iterations of the model. In each, one value was randomly sampled from every distribution and those values were used to calculate costs of interventions over the planning horizon.

**Table 1:** Parameters used in simulated intervention costs.

Parameter	Description	Value(s)	Units	Sources/notes
T	HCW hand touches per hour on mobile phones while on duty	Uniform(2.41, 2.95)	#	±10% of the estimate of Zhang et al. (40) (taken in an office setting).
$D_{\mathrm{sw}}$	Duration of a hand wash with soap and water	Uniform(15.93, 19.47)	s	±10% of the estimate of Sayeed et al. (41).
F	Tap flow rate	0.33	L/s	Water Corporation (42).
$P_{w}$	Water price	2.78 × 10^−3^	$/L	Water Corporation (43).
$\alpha_{1}$	Proportion of HCW who routinely disinfect their phone using other methods (e.g., alcohol wipes) while on duty	Uniform(0.12, 0.14)	#	±10% of the estimate of Cavari et al. (44).
$V_{s}$	Volume of (liquid) soap used per handwash	4	ml	Schwarcz (45) estimate 2.3 g, equivalent to a volume of ≈4 ml.
$P_{s}$	Price of soap	0.03	$/ml	Alpha Medical Solutions (46).
$V_{h}$	Volume of hand sanitizer used per handwash	4	ml	Zingg et al. (47).
$P_{h}$	Price of hand sanitizer	Uniform(0.005, 0.01)	$/ml	Assumes the hospital purchases hand sanitizer at 25%–50% of the price paid by a single consumer (48).
$V_{t}$	Volume of paper towel used per handwash	Discrete(1, 2, 3)	Sheets	Plausible value range.
$P_{t}$	Price of paper towels	0.04	$/sheet	Medical Search Australia (49).
W	Wage rate	Uniform(28.53, 63.33)	$/h	Fair Work Ombudsman (50).
$\alpha_{2}$	Ratio of hand washes with hand sanitizer to washes with soap and water	0.25	#	Assumes four washes with hand sanitizer for every one wash with soap and water.
$D_{h}$	Duration of a hand wash with hand sanitizer	Uniform(18, 22)	s	±10% of the estimate of Centers for Disease Control and Prevention (24).
B	Number of patient beds in the hospital	200	#	Plausible value.
$\alpha_{3}$	HCW-to-patient ratio	0.18	#	Assumes $\partial_{3}=\frac{1}{4}$ during day shifts (12 h) and $\partial_{3}=\frac{1}{7}$ during night shifts (51, 52).
r	Discount rate	Uniform(0.03, 0.07)	#	Parliament of Australia (53).
E	Power rating of a standard UV-C sanitization device	250	W	Plausible value.
$D_{\mathrm{uv}}$	Duration of mobile phone sanitization using a UV-C sanitization device.	10	s	Glissner (54).
$P_{e}$	Price of electricity	33	$/kWh	Calma (55).
$P_{\mathrm{uv}}$	Purchase and installation price of a single UV-C sanitization device	Uniform(2,000, 2,500)	$	Plausible value range.
O	Operational life of a UV-C sanitization device	Uniform(5, 7)	Years	Plausible value range.
$\alpha_{4}$	Maintenance cost of a UV-C sanitization device expressed as a proportion of the purchase and installation costs	Uniform(0.05, 0.15)	#	Plausible value range.
n	Planning horizon	10	Years	Plausible value.
$\alpha_{5}$	UV-C sanitization device to hospital bed ratio	0.5	#	Plausible value.

## 3 Results

**Figure 1:**
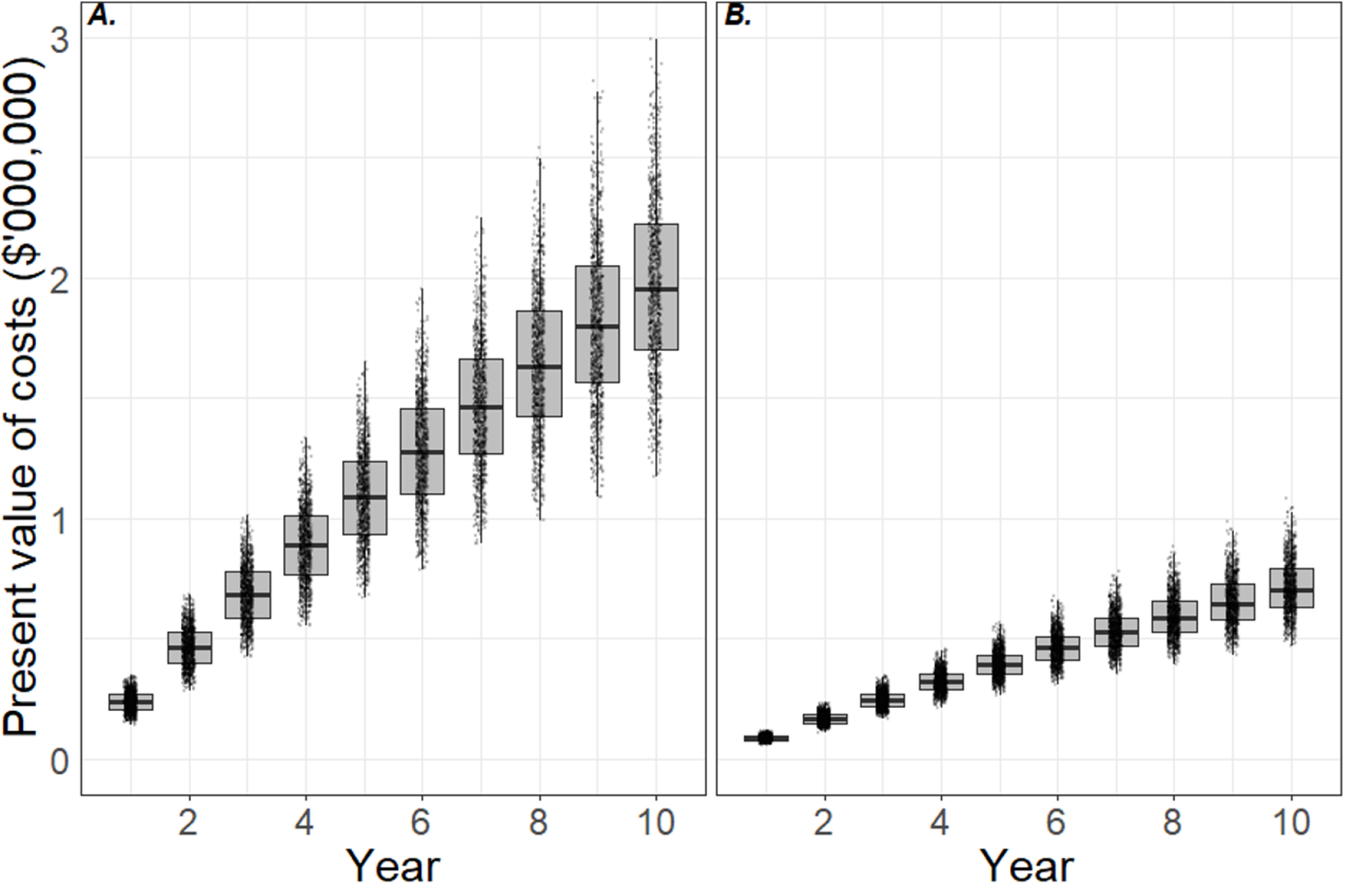
Cost effectiveness of interventions to minimize health care-associated infection risks due to mobile phones. Panels **A** and **B** show the present value of costs incurred by the hospital using hand sanitization protocols and ultraviolet-C sanitization devices, respectively. The plots indicate the 5th, 25th, median, 75th and 95th percentiles and simulation data are superimposed. By year 10, the hospital is likely to have outlaid $1.7–2.2 million (5th–75th percentiles) in material and opportunity costs by adopting hand sanitization protocols (panel **A**), and $0.6–0.8 million by adopting ultraviolet-C sanitation devices (panel **B**). This is equivalent to $850–1,100 per bed per year and $300–400 per bed per year, respectively.

The present value of costs incurred by the hospital to manage HAI risks from mobile phones over a 10-year period are shown in Figure 1. Panel A shows the costs of using hand washing protocols and panel B shows the costs of using UV-C mobile phone sanitization devices. The boxplots show the 5th, 25th, median, 75th and 95th percentiles of costs predicted in the model over 10 years. By 10th year, 50% of the model iterations predicted cumulative costs of between $1.65 million and $2.18 million if the hospital used hand washing protocols alone, with median costs of $1.93 million (panel A). This equates to a real or present value of cost per bed of $965 per year, 74% of which were material costs and 26% opportunity costs. In contrast, cumulative costs of using UV-C sanitization devices over the same period were between $0.64 million and $0.79 million, with a median cost of $0.72 million. This equates to around $360 per bed per year in real terms, of which 46% were material costs and 54% opportunity costs.

**Figure 2:**
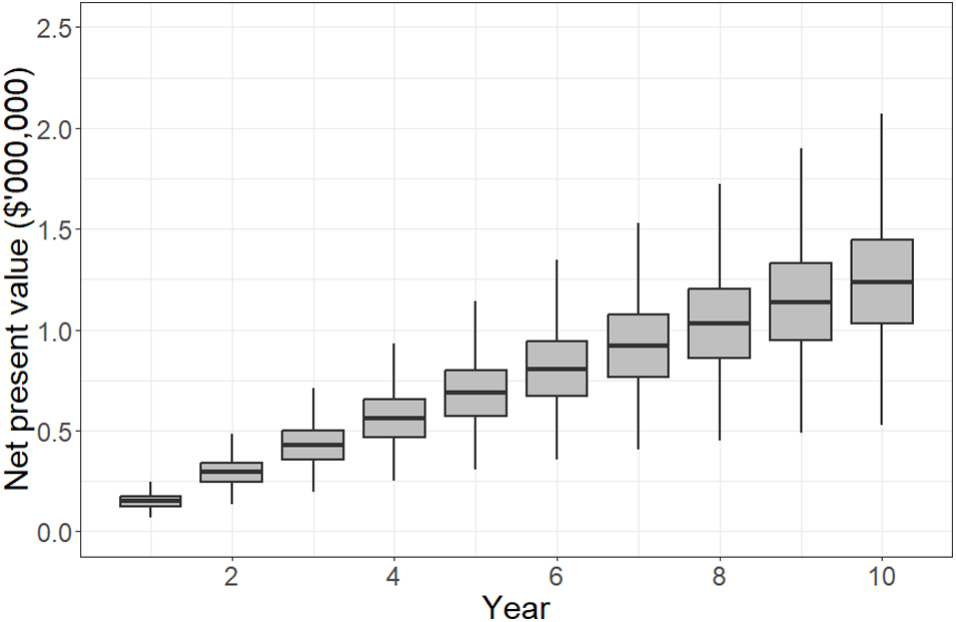
Net present value accruing to the hospital over a 10-year planning horizon if ultraviolet-C sanitization devices are used to minimize health care-associated infection risks due to mobile phones. The plots indicate the 5th, 25th, median, 75th and 95th percentiles. By year 10, results show that the hospital is likely to have gained $1.0–1.4 million (5th–75th percentiles) due to avoided costs related to hand hygiene practices, which is equivalent to $500–700 per bed per year.

The net present value to the hospital from investing in UV-C mobile phone sanitizers is plotted in Figure 2. These are effectively the hand sanitization costs avoided by using UV-C sanitizers instead. After 10 years, 50% of model iterations showed a net present value of between $1.01 million and $1.45 million. The median net present value accrued by the hospital was $1.21 million, which equates to $605 per bed per year if one device is installed for every two beds.

**Figure 3:**
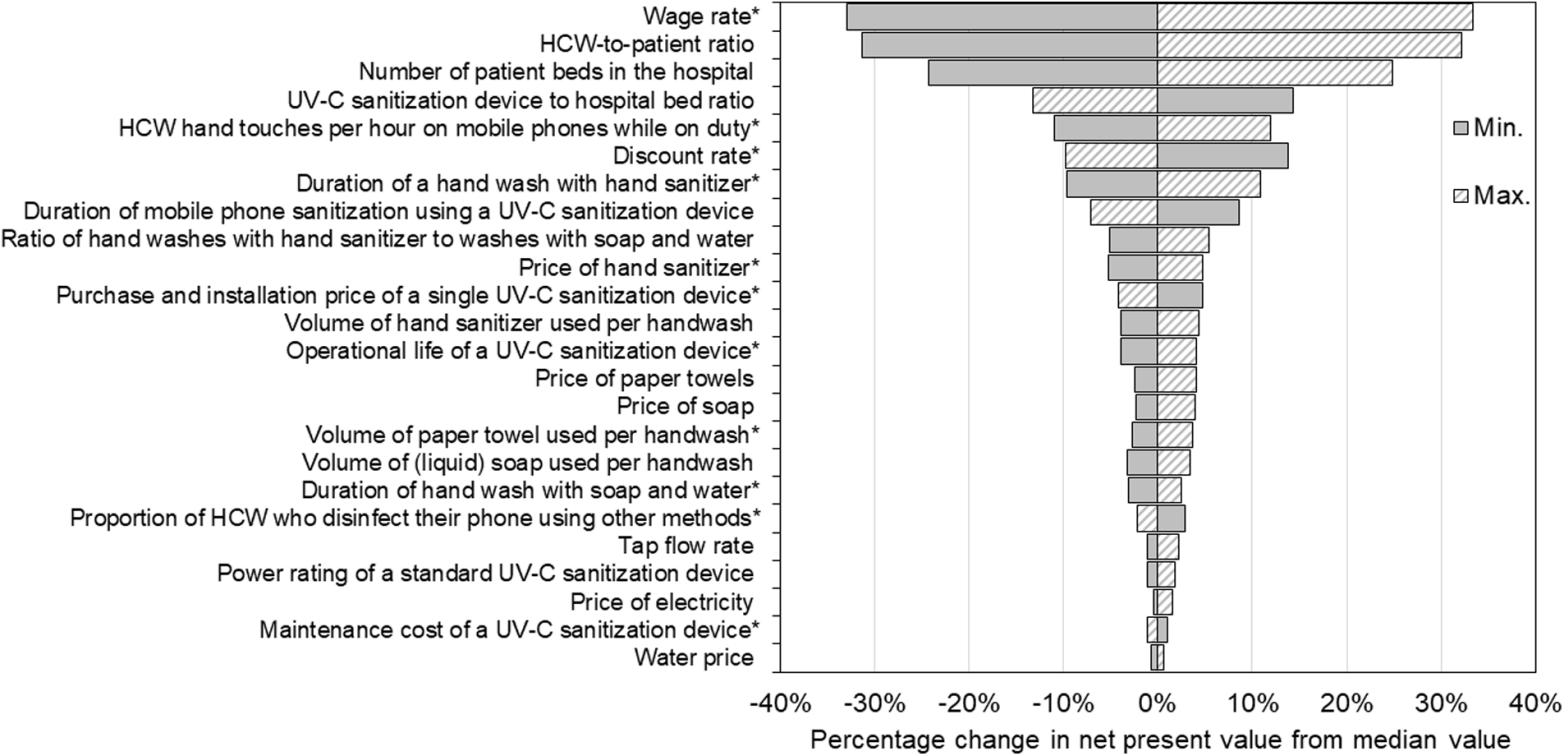
Sensitivity of net present value results to changes in parameter values. Parameters are ranked from top to bottom according to the sensitivity of the net present value over 10 years to changes in each, with the length of the bars corresponding to each parameter reflecting the change in results produced. Each parameter specified as a distribution (indicated by *) was changed to the minimum and maximum values of its range while all other parameters remained unchanged, and the resultant net present value recorded. Parameters specified as fixed values were changed by ±25%. The net present value was most sensitive to changes in the wage rate, healthcare worker-to-patient ratio, the number of beds in the hospital and the number of ultraviolet-C devices per bed.

Given the uncertainties in many parameters, we carried out a sensitivity analysis to determine which had the greatest influence on the results. We used a simple approach whereby each parameter was set to the minimum and maximum values of its range (in the case of parameters specified as distributions), or by ±25% (in the case of fixed value parameters) while all other parameters remained unchanged. The influence the minimum and maximum values of each parameter had on the net present value to the hospital over 10 years is reported in Figure 3. Here, the sensitivity of results is indicated by the length of bars corresponding to each parameter, and parameters are ranked from top to bottom in order of the change they produced in net present value. Parameters that were specified as distributions (Table 1) are indicated with asterisk.

Our results were most sensitive to changes in the wage rate as the duration of hand washing was longer than UV-C phone sanitization, causing greater opportunity costs. As such, wages had a strong positive relationship with net present value for our hypothetical 200-bed hospital, meaning that as wages were increased the return on investment in UV-C mobile phone sanitizers also increased, and vice versa. Raising the wage rate from its median value of $45.90/h to $63.30/h (i.e., +38%) produced a proportional rise in net present value from $1.21 to $1.61 million (+33%) over 10 years, and decreasing wages to $28.50/h (−38%) lowered net present value to $0.81 million (−33%).

The second most sensitive parameter was the HCW-to-patient ratio. This parameter also had a positive relationship with the return on investment in UV-C sterilizers with higher (lower) HCW-to-patient ratios increasing (decreasing) the net present value. Increasing the HCW-to-patient ratio to 0.22 (i.e., +25% from its median value) increased the net present value to $1.60 million (+32%). Conversely, lowering the HCW-to-patient ratio to 0.14 (−25%) lowered the net present value to $0.84 million (−31%).

The number of beds in the hospital and the number of UV-C devices per bed also produced large changes in the net present value. Increasing the number of beds in the hospital by ±25% produced directly proportional changes in the net present value as we assumed constant marginal (i.e., per unit) costs of UV-C devices. In contrast, the deployment rate of UV-C devices was inversely related to net present value as we assumed each intervention is equally effective at reducing HAI risks. Increasing the number of UV-C devices per bed from 0.5 (or one device for every two beds) to 0.625 (or five devices for every eight beds) lowered the net present value to $1.06 million (−13%), and decreasing the number of devices per bed to 0.375 (or three devices for every eight beds) increased the net present value to $1.40 million (+14%).

Results were also relatively sensitive to the number of times per hour HCW touched their phones, specified as Uniform (2.41, 2.95). Changes in the value of this parameter were positively related to net present value over the 10-year planning horizon, producing an 11% fall in net present value (to $1.08 million) using the minimum value and a 12% increase in net present value (to $1.37 million) using the maximum value.

## 4 Discussion

Of the two interventions we considered, our results showed hand hygiene protocols to be the least cost-effective option for controlling HAI risks from mobile phones. The present value of median hand sanitization costs was approximately $965 per bed per year after 10 years compared to $360 per bed per year for UV-C sanitizers. This implies that hospital managers can potentially save $605 per bed per year by adopting UV-C mobile phone sanitizers rather than hand washing protocols, or a total of $0.12 million annually in a 200-bed hospital.

The most important determinant of costs saved is the rate of pay of the HCW who will use the devices while on duty. The range of wages we used in the simulation reflect the minimum and maximum wages received by registered nurses stated in the Australian Nurse Award Pay Guide (50). As such, we have probably underestimated wages and opportunity costs resulting from each intervention as we do not account for specialized HCW receiving higher wages, or HCW working on weekends or on public holidays. The wage bill of each hospital will also vary according to the mix of HCW employed, hospital specialty units and the infrastructure needed to support these.

The return on investment in UV-C mobile phone sanitizers will be higher in hospitals with relatively high HCW-to-patient ratios. In hospitals with higher HCW-to-patient ratios patients tend to experience better health outcomes (51), and fatigue and job dissatisfaction amongst HCW are also lower (56). But to attain these benefits hospitals must incur higher wage bills. We suggest that the benefits of higher HCW-to-staff ratios could be enhanced by hospital managers investing in UV-C devices to manage HAI risks from mobile phones instead of using conventional handwashing protocols. Our base assumption was that the hospital would need to provide one UV-C device for every two patient beds to achieve the same reduction in risk as handwashing protocols, but if this risk reduction could be achieved with a smaller number of devices the hospital's return on investment will be higher.

It is clear from our results that the cost advantage of UV-C devices over conventional hand hygiene protocols will increase as mobile phones become more integrated into the workplace. The minimum and maximum bounds we used to simulate the number of times HCW touched their phones were based on the estimate of Zhang et al. (40), who studied workers in an office rather than in a hospital setting, so we may have over or underestimated this parameter. Further research is needed to better understand mobile phone use patterns in hospitals as the number of medical applications made available on mobile devices rises (57, 58) and their use as point-of-care tools is actively encouraged (59, 60). Our findings suggest that this trend will increase returns on investments in UV-C phone sanitization technologies.

There are several practical issues hospital managers must consider in addition to the tangible return on investment when deciding whether to invest in UV-C devices. This technology only addresses one potential source of HAIs (i.e., residual surface contamination of mobile phones). Multiple other potential modes of transmission will not be mitigated (61). Some investigation is also required when deciding which devices to buy as their suitability for hospital environments varies. For example, some require a door to be opened and closed as part of the operating procedure, introducing the possibility of HCW's hands becoming (re)contaminated (34). The size of mobile devices to be accommodated by UV-C devices is not consistent, with larger devices like tablets now commonly used as patient-bound devices (34). Frequent exposure to UV-C over time may also cause damage to the materials devices are constructed from (28), so it may be necessary to use protective covers. However, these pose a greater cross-infection risk than flat screen surface as they can potentially harbor more microorganisms (30). We did not account for any of these considerations in our analysis.

We also assumed that the costs of training staff in the correct use of UV-C devices would be minimal as they are automatic, easy to operate and, unlike automated hand hygiene technologies, do not require dedicated staff to oversee their use (54, 62). Automatic reminders on mobile devices can provide a standardized schedule to perform disinfections regularly (30, 63), and some UV-C devices make use of built-in dose monitoring systems that measure the UV-C dose given during the disinfection cycle (28, 64). There may be value in training staff on some of the benefits of UV-C technology to ease its integration into the workplace, such as shorter disinfection time and reduced cost of chemical disinfectants. In some hospital rooms with limited space, particularly those needing bulky specialist equipment, there could be limited space to introduce new apparatus. However, UV-C devices designed specifically to sanitize mobile phones tend to be small and can be mounted on walls so as not to form obstructions.

Further investigation is needed to estimate environmental costs associated with interventions to minimize HAI risks from mobile phone use. No method of hand washing is environmentally benign. Hand sanitization using isopropanol-based products has been shown to have a lower overall environmental impact than alcohol-based products or soap and water but is relatively intensive in terms of fossil fuel inputs (65). Carbon emissions resulting from water heating for handwashing, ostensibly to achieve greater effectiveness, may exceed the total emissions from many industrial sources, including the lead and zinc industries (66). To include these costs into our assessment would require data showing the proportion of isopropanol-based and ethanol-based hand sanitizers and the proportion of facilities using warm/hot water for soap-based hand washes. Information revealing the resources used in constructing and maintaining UV-C phone sanitizers is also needed to undertake a comparative life cycle assessment for each intervention, and to explore how their environmental footprint might be changing as the technology evolves. For instance, the latest generation of UV-C devices do not use mercury-vapor lamps, which might make them more environmentally friendly than older devices (28, 67).

We have assumed that UV-C phone sanitizers and handwashing protocols are alternative strategies hospitals can use to mitigate HAI risks associated with mobile phones, but in practice they are not mutually exclusive. Indeed, they could be viewed as complimentary. In some ways, mobile phones have become extensions of HCW hands (13), and despite efforts of the World Health Organization and other public health authorities to promote active hand hygiene, mobile phones could negate hand hygiene protocols if they are treated separately (36). If instead UV-C phone sanitization was coupled with hand hygiene to prevent the dynamic cross-contamination between hands and mobile phones, and vice versa, hospitals could achieve a maximum reduction in phone-related HAI risk (13, 21). However, this may or may not be optimal in terms of satisfying the financial objectives of hospital managers, and would need to be determined on a case-by-case basis.

## 5 Conclusion

Hand hygiene is vital for the prevention of microbial dissemination in medical wards. Mobile phones are increasingly integrated into healthcare and their use is now an important consideration for hospital managers implementing HAI risk management strategies. These high touch devices are known to harbor viable microbes and negate the benefits of hand hygiene protocols unless properly sanitized. In this paper we looked at two strategies hospital managers could use to minimize HAI risks associated with mobile phones with the objective of identifying which involved the lowest cost. One strategy used hand hygiene protocols requiring HCW to sanitize their hands after using their mobile phones and before attending to patients. This could involve the use of liquid hand sanitizers or soap and water. A second strategy used UV-C phone sanitizers to treat HCW phones after each use. If these options are considered alternatives, rather than compliments, we showed that over a 10-year period the strategy using UV-C phone sanitizers was approximately $500–700 per bed per year cheaper than using hand hygiene protocols. We estimated that a 200-bed hospital could save $1–1.4 million over a 10-year period if managers were to choose this option providing one UV-C device for every two patient beds. Our results were sensitive to changes in the HCW-to-patient ratio, the wage rate, the number of mobile phone touches per hour by HCW and the rate of deployment of UV-C sanitizer units per bed. Our analysis did not include practical aspects of installing UV-C devices, such as training needs, potential damage to mobile devices after repeated exposure to UV-C and space requirements. We also did not include environmental costs of either intervention. While the results of our hypothetical study were positive, further research is warranted to explore the feasibility and long-term implications of adopting UV-C mobile phone sanitization technologies on a broad scale.

## Data Availability

The raw data supporting the conclusions of this article will be made available by the authors, without undue reservation.
